# The Evaluation of UPro as a New Nutrient on High-Quality Egg Production From the Perspective of Egg Properties, Intestinal Histomorphology, and Oviduct Function of Laying Hens

**DOI:** 10.3389/fnut.2021.706067

**Published:** 2021-08-20

**Authors:** Xinyu Chang, Kai Qiu, Jing Wang, Haijun Zhang, Shizhou You, Shuichao Mi, Guanghai Qi, Shugeng Wu

**Affiliations:** ^1^Risk Assessment Laboratory of Feed Derived Factors to Animal Product Quality Safety of Ministry of Agriculture and Rural Affairs, National Engineering Research Center of Biological Feed, Institute of Feed Research, Chinese Academy of Agricultural Sciences, Beijing, China; ^2^Changzhou Yayuan Biochemical Technology Co., Ltd, Jiangsu, China

**Keywords:** UPro, egg quality, intestinal histomorphology, oviduct function, laying hens

## Abstract

This study was to investigate the effects of UPro as a new nutritive fortifier on high-quality egg production from the perspective of egg properties, intestinal histomorphology, and oviduct function of laying hens. Four hundred thirty-two Hy-Line Brown laying hens aged 56 weeks were allocated to four groups. Layers were given a basal diet or supplemented with different levels of small peptides (0.2, 0.4, and 0.8%) to replace soybean meal. After 1-week adaptation period, the feeding trial was conducted for 12 weeks. The results showed that UPro addition significantly decreased (*P* < 0.05) the hardness, stickiness, and chewiness of albumen of layers on weeks 12. A linear elevation (*P* < 0.05) in the albumen height, Haugh unit (HU), and crude protein content of albumen of layers were noted on week 12 along with dietary UPro addition increasing, and the villus height (VH) and villus height-to-crypt depth radio (VCR) of jejunum also linearly increasing (*P* < 0.05). In addition, there were linear elevations (*P* < 0.05) in the relative mRNA expression of Sec23 homolog A (*Sec23A*) and protein-O-mannosyltransferase1 (*POMT1*) in layers as dietary UPro addition increased. In conclusion, dietary UPro addition could improve intestinal health, increase the absorption of nutrients, and improve egg quality of laying hens. The possible mechanism underlying UPro improving the quality and processing characteristics of albumen is up-regulating *Sec23A* and *POMT1* expression of magnum. These findings will promote the application of UPro as a new nutritional additive in the production of high-quality eggs.

## Introduction

Eggs are an important source of animal protein that are rich in high-quality protein and essential unsaturated fatty acids, and are easy to obtain for humans (1). Compared with eggs, there are no other animal-derived foods that can be cooked in various ways (2). Protein-sourced feed plays a vital role in regulating and maintaining the quality of eggs (3, 4). Therefore, enhancing the protein nutrition of laying hens is needed for the production of high-quality eggs.

UPro, a functional and nutritional additive synthesized from corn, is rich in bioactive substances in the form of small peptides, free amino acids, and a small amount of lactic acid. Small peptides are composed of two or three amino acids, also called oligopeptides, small molecule active peptides, or short peptides (5). In addition to free amino acids, the final products of proteins in the digestive tract also had some small peptides, which demonstrated that short peptides could be absorbed in the form of dipeptides and tripeptides and enter the blood circulation (6–8). Small peptides transport was more effective than the same level amino acids (9, 10). Researchers have found that small peptides have various physiological functions for animals (11, 12). Nowadays, the application of small peptides as feed additives to diets has an essential role in promoting the production performance of animals, such as improving the production performance of dairy cows, piglets, and broilers (13–15), which can also ameliorate the intestinal flora distribution of suckling pigs (16). Small peptides were also considered to have antibacterial and antiviral properties, which could accelerate the growth of beneficial bacteria in the intestines and improve the digestion and absorption of livestock and poultry. For instance, the skin of forest frogs could secrete a number of bioactive peptides with simple structure, which could inhibit gram-positive and gram-negative bacteria (17, 18); the cecropin antimicrobial peptide could improve the intestinal absorption of piglets (19). Thus, it could be hypothesized that UPro enriched with small peptides probably have good application prospects to strengthen protein nutrition in livestock and poultry production.

Nowadays, the quality of albumen of eggs on the market is unable to satisfy the increasing level of demand. It was well demonstrated that the source and quality of protein feed are closely related to albumen quality of eggs (20). However, the mechanism underlying how dietary protein nutrition interacts with the egg quality in laying hens is still unclear. The small peptides are widely acknowledged to be implicated in the regulation of protein synthesis, secretion, and glycosylation (21, 22), which are three important processes of albumen formation (21, 23). It is meaningful to determine the expression of genes related to the synthesis [*OVOA, OVOB, OVALX*, and *OVAL*, (24)], secretion [*Sec13* and *Sec23A*, (25)], and glycosylation [*POMT1* and *POMT2*, (26)] of protein in magnum to explore the possible mechanism of UPro regulating albumen quality. For the purpose of improving albumen quality, the current study aimed to explore the effects of dietary UPro supplementation on the performance, egg quality, and protein gel properties in laying hens and clarify its possible mechanism through oviduct function.

## Materials and Methods

### Birds and Housing

Animal care and handling in this experiment was supported by the Animal Care and Use Committee of the Feed Research Institute of the Chinese Academy of Agricultural Sciences, Beijing. The approval number of animal ethics is CAAS.No20200507S0600103.

A total of 432 Hy-Line Brown laying hens aged 56 weeks were allocated to one of four dietary treatments. Each group had six replicates (18 birds per replicate). Three birds were housed in one cage (40 × 40 × 35 cm). Medium-sized layer cages used in the experiment were three layers with a fully stepped assembly method. The cage system included a water tub composed of a water tank and a nozzle, and a fodder tub. Birds received 16 h of light every day and the light value was controlled at 20Lux. The temperature of the henhouse was maintained at 24°C. The humidity of the henhouse was controlled at 50–80%. Feed and fresh water were provided *ad libitum*.

### Test Material and Experimental Design

UPro creation method used in this study is based on the research of Wang et al. (27). Specifically, it was made from corn seeds as the primary raw material, through soaking, concentration, hydrolyzing by trypsin and pepsin, implanting in Bacillus subtilis, spray drying, powder coating, and physical cooling. It was determined that the UPro contains 66.78% of peptides and 9.80% of free amino acids, containing 26% lactic acid. UPro has the functions of biologically active peptides, such as lowering blood pressure, detoxifying alcohol, and being an antioxidant (28, 29). The composition and nutritional level of the basal diet based on corn-soybean meal for layers are presented in Table 1. The control group was given the basal diet. The other three groups were given the diets 0.2, 0.4, or 0.8% soybean meal of the basal diet replaced by UPro, respectively. All layers were fed the basal diet for 1 week and the diets of the experimental groups were gradually replaced with the trial feeds. Samples were collected after the 56-week-old laying hens had passed the adaptation period.

**Table 1:** Dietary composition and nutrient levels of the basal diet.

**Ingredient**	**%**	**Nutrient level^**a**^**	**%**
Corn	61	Crude protein	16.5
Soybean meal	23.86	Calcium	3.5
Limestone	8.9	Total phosphorus	0.6
Bran	3.35	Available phosphorus	0.32
Soybean Oil	1.2	AME (MJ/kg)	11.29
Dicalcium phosphate	0.9	Lysine	0.75
Choline chloride	0.12	Methionine	0.34
DL-methionine	0.12	Methionine + cysteine	0.65
Phytase	0.05		
Antioxidants (VE)	0.02		
Experimental premix^*b*^	0.35		
Vitamin and mineral premix^*c*^	0.13		
Total	100		

a *Experimental diets were formulated according to the NRC requirements 1994 (30) and meet nutrients recommendations of Chinese Feeding Standard of Chicken (NY/T 33-2004) (31).*

b *Vitamin and mineral premix provided the following per kg of diets: VA, 12,500 IU; VD3, 4,125 IU; VE, 15 IU; VK, 2 mg; VB1, 1 mg; VB2, 8.5 mg; VB6, 8 mg; VB12, 5 mg; calcium pantothenate, 50 mg; niacin, 32.5 mg; biotin, 2 mg; folic acid, 5 mg; choline, 500 mg; Mn, 65 mg; I, 1 mg; Fe, 60 mg; Cu, 8 mg; Zn, 66 mg.*

c *Includes NaCl, Na_2_SO_4_*.

### Laying Performance Parameters and Egg Quality

The number of eggs and the total weight of eggs were recorded daily. The feed consumption was recorded biweekly, and the value of average daily feed intake (ADFI) was calculated. The feed conversion ratio (FCR) was calculated by dividing ADFI by egg weight. The percentage of egg production was calculated weekly. Egg mass was calculated by multiplying egg weight by egg production. At the end of week 4, 8, and 12, five eggs per replicate were collected to assess egg quality. The egg shape index was calculated by dividing the long axis (cm) by the short axis (cm). Eggshell thickness was measured with an Eggshell Thickness Gauge (Orka Technology Ltd, Ramat Hasharon, Israel). Eggshell strength was measured with an Egg Force Reader (Orka Technology Ltd). Egg strength and thickness were carried out under fresh shell conditions without any prior treatments. The egg weight, albumen height, yolk color, and HU were measured using an Egg Analyzer (Orka Technology Ltd).

At the end of week 12, the day after measuring egg quality, six eggs of each replicate were collected, three of which were for determining albumen weight, albumen proportion, and the water or crude protein contents of albumen, and the other three were used to measure the weight of thick albumen. Eggshell was also weighed after drying. The albumen was put in a disposable petri dish and stored in the refrigerator at −20°C for freeze-drying. Albumen weight was calculated by deducting the weight of yolk and shell from the egg weight. Albumen proportion was calculated as albumen weight divided by the whole egg weight. The albumen samples were freeze-dried using LGJ-12 Vertical Freeze Dryer (Nanbei Instrument Equipment Co., Ltd, Zhengzhou, China) and the content of water in albumen was calculated by dividing the weight difference before and after the lyophilization of the egg white by the weight of the egg white. The content of crude protein in albumen was analyzed using Kieldahl Azotometer (C. Gerhardt GmbH Co., Ltd, Mexico, Germany).

### Protein Gel Properties

At the end of week 12, one egg from each replicate was collected to assess protein gel properties. The gel properties of egg white were determined according to the method of Houška et al. (32). The albumen separated from the egg was evenly mixed, and 15 mL of them was sucked by a syringe, slowly injected into the 25 mL small beaker, and finally stored in the refrigerator at 4°C for 12 h. The beakers were sealed with tin foil and put into a water bath thermostat at 100°C for 30 min. Next, samples were immediately put into cold water to cool. The heat-induced albumen was cut into small squares with the size of 1 cubic centimeter for measuring the protein gel properties. Hardness, cohesiveness, springiness, gumminess, and chewiness were measured using a Food Technology Corporation in a texture analyzer (Food Technology Corporation, Mecmesin Ltd, Brighton, England).

### Blood Sampling and Laboratory Analyses

At the end of week 4, 8, and 12, one bird per replicate was selected randomly. Blood samples (3 mL) were taken from the wing vein by the laboratory animal practitioners. The process conformed to the regulations of animal ethics and the sample was centrifuged at 3000 × g for 10 min to isolate serum. The centrifuge temperature was set to 4°C. Then, serum was kept in the refrigerator at −20°C immediately. The blood was in a low temperature environment during the whole process, which ensured that the enzyme was not be inactivated. The concentrations of total bilirubin (TB), alanine aminotransferase (ALT), aspartate aminotransferase (AST), alkaline phosphatase (ALP), Total protein (TP), albumin (ALB), glucose (GLU), and uric acid (UA) in serum were analyzed using an automatic biochemical analyzer (Model 7020, Hitachi, Tokyo, Japan).

### Histomorphometry of Intestine

The birds selected for blood sampling at week 12 were then executed and dissected. About 3 cm of the middle section of jejunum and ileum were fixed in 10% formaldehyde solution, embedded in paraffin, cut into 5 μm sections, and then stained with hematoxylin-eosin for histological analysis. An Olympus BX43 microscope (Olympus Corp., Tokyo, Japan) was used to measure the villus height (VH) and crypt depth (CD). The villus height-to-crypt depth radio (VCR) was calculated by dividing VH by CD.

### mRNA Extraction, cDNA Synthesis, and Quantitative Real-Time PCR

Total mRNA was extracted from magnum tissue of fallopian tube using EasyPure® RNA Kit (Trans Gen Biotech Co., Ltd, Beijing, China) following the manufacturer's protocol. The cDNA samples were synthesized from the 1000 ng of total mRNA using TransScript® RT/RI Enzyme Mix (TransGen Biotech Co., Ltd, Beijing, China). The synthesized cDNA was stored in a refrigerator at −20°C.

Quantitative real-time PCR (qPCR) was performed with the SYBR® Fast Universal qPCR Kit (QIDI TAILI Technology Co., Ltd, Beijing). The gene relative expression was measured using an iCycler iQ multicolor real-time PCR system (Bio-Rad Laboratories, Hercules, CA, USA). β-Actin was used as the internal reference gene for normalization. Primer sequence of β-actin and the target genes, including *OVOA, OVOB, OVALX, OVAL, Sec13, Sec23A, POMT1* and *POMT2*, are shown in Table 2. The cycling protocol was made as follows: 95°C for 5 min, 40 cycles of 95°C for 10 s, 60°C for 30 s. For the calculation of the relative expression value of genes, we referred to the research of Livak and Schmittgen, which described in detail how to use the 2^−Δ*ΔCt*^ method to calculate the relative expression of genes (33).

**Table 2:** Information for primer sequences.

**Primers^**a**^**	**Sequence (5^**′−3′**^)**	**Tm (^**°**^C)**	**Product size (bp)**
*β-Actin*	F: GAGAAATTGTGCGTGACATCA	56	152
	R: CCTGAACCTCTCATTGCCA		
*OVOA*	F: TGTGTTTCTGTCTGCGGTTG	56	182
	R: ATTTTGCGTACGAAGCCCTC		
*OVOB*	F: TGCGTCTACCAAGCCTGTAA	56	177
	R: TGCCTGAGTGTTGTAGCTGA		
*OVALX*	F: AGTGTACCTGCCCCAAATGA	56	192
	R: CCTGCCATCTCAATGCCATC		
*OVAL*	F: CCCCATTGCCATCATGTCAG	56	225
	R: AGTCTACTGGCAAGGCTGAA		
*Sec13*	F: CTGGACATGCGATGATGCCTCTG	61	238
	R: CTGCTCATTCTGCTGCCCTTCTG		
*Sec23A*	F: TGGACCCTGCGTATCTCTGAACTC	61	357
	R: CCCTCTTCTGTCTCCGCCCTATAC		
*POMT1*	F: CTGGTCCACGGCATCACAACTC	60	327
	R: TTCCACATTCCACACCATGCTCTG		
*POMT2*	F: GCCTCATCTTGCTTCCGCTCAC	61	375
	R: CTCCATGCCTCACAAACTCCACAG		

a *F, forward; R, reverse*.

### Statistical Analysis

Data was processed by one-way ANOVA program of the SPSS 19.0 (SPSS Inc., Chicago, IL, USA). The mean of the replicate served as an experimental unit of parameters. Besides, the linear and quadratic responds against UPro dose were evaluated using the polynomial regression analysis, which could apply to all variables. Differences among treatments were assessed using Duncan's multiple comparisons. Intestinal histomorphometry and gene expression were also assessed by ANOVA and Duncan's multiple comparisons. *P* < 0.05 was considered to be significant and 0.05 ≤ *P* < 0.10 was defined as a trend of significance.

## Results

### Performance and Egg Quality

UPro addition had no effect (*P* > 0.05) on egg production, average egg weight, egg mass, ADFI, and FCR of laying hens during the experimental period (Table 3). The effects of UPro levels on egg quality are displayed in Table 4. No significant differences were observed in egg weight, egg shape index, eggshell thickness, eggshell strength, and yolk color between treatments at week 4, 8, and 12 (*P* > 0.05). However, albumen height and HU were linearly affected by UPro supplementation in diets at week 8 (*P* < 0.05). Albumen height and HU of albumen showed linear and quadratic increases along with the increase of dietary UPro addition at week 8 and 12 (*P* < 0.05). Among the treatments, no significant changes (*P* > 0.05) were detected in albumen weight, albumen proportion, and the water content of albumen at week 12 (Table 5). Compared with the control, the crude protein content of albumen of birds fed diets with 0.4 or 0.8% UPro significantly increased (*P* < 0.05) and also showed linear and quadratic increases along with the increasing UPro addition (*P* < 0.05).

**Table 3:** Effects of dietary UPro on production performance of laying hens.

**Items^**a**^**	**Time (w)**	**UPro levels (% intake)**				**SEM**	***P*** **-value** ^**b**^		
		**0**	**0.2**	**0.4**	**0.8**		**A**	**L**	**Q**
Egg production (%)	1–4	87.57	89.65	86.84	87.63	0.43	0.11	0.36	0.66
	5–8	86.44	88.03	84.86	86.90	0.74	0.52	0.90	0.90
	9–12	82.54	85.82	81.72	83.40	0.70	0.19	0.92	0.99
	1–12	85.52	87.83	84.47	86.03	0.56	0.20	0.72	0.91
Egg weight (g)	1–4	61.00	61.89	61.76	62.19	0.23	0.30	0.84	0.98
	5–8	61.58	61.82	62.37	62.02	0.36	0.90	0.64	0.78
	9–12	61.12	61.69	60.89	61.57	0.25	0.67	0.75	0.92
	1–12	61.23	61.80	61.69	61.93	0.24	0.77	0.73	0.90
ADFI (g)	1–4	111.13	111.75	112.70	110.85	0.60	0.74	0.85	0.56
	5–8	113.80	112.79	116.30	113.12	0.94	0.57	0.62	0.62
	9–12	106.31	106.86	105.45	105.98	0.61	0.89	0.24	0.50
	1–12	110.41	110.46	111.49	109.98	0.60	0.86	0.40	0.68
Egg mass	1–4	54.38	55.59	53.29	54.11	0.42	0.30	0.49	0.77
(g/hen/day)	5–8	53.23	54.42	52.91	53.87	0.53	0.78	0.89	0.99
	9–12	50.46	52.95	49.76	51.40	0.46	0.07	0.98	1.00
	1–12	52.70	54.31	51.66	53.12	0.42	0.17	0.84	0.92
FCR	1–4	2.08	2.02	2.10	2.04	0.02	0.19	0.68	0.60
	5–8	2.14	2.07	2.20	2.10	0.02	0.30	0.56	0.62
	9–12	2.11	2.02	2.12	2.06	0.02	0.09	0.21	0.44
	1–12	2.11	2.04	2.14	2.07	0.02	0.09	0.54	0.70

a *ADFI, average daily feed intake; FCR, feed conversion ratio (feed: egg, g: g).*

b *P-value: A represents one-way ANOVA and Duncan's multiple comparisons; L and Q represent linear and quadratic analysis using regression analysis, respectively*.

**Table 4:** Effects of dietary UPro on egg quality of laying hens.

**Items^**a**^**	**Time (w)**	**UPro levels (% intake)**				**SEM**	***P*** **-value** ^**b**^		
		**0**	**0.2**	**0.4**	**0.8**		**A**	**L**	**Q**
Egg shape index	4	1.32	1.31	1.33	1.32	0.004	0.25	0.46	0.65
	8	1.33	1.33	1.33	1.32	0.003	0.98	0.75	0.93
	12	1.32	1.32	1.33	1.31	0.002	0.16	0.14	0.10
Eggshell	4	0.46	0.46	0.45	0.45	0.003	0.72	0.46	0.46
Thickness (mm)	8	0.45	0.45	0.44	0.45	0.002	0.47	0.36	0.58
	12	0.44	0.44	0.45	0.45	0.002	0.18	0.07	0.19
Eggshell	4	36.69	37.36	35.39	36.99	0.58	0.68	0.97	0.83
Strength (N/m^*b*^)	8	36.68	36.44	35.51	36.54	0.54	0.88	0.90	0.78
	12	36.06	33.35	35.21	34.64	0.75	0.65	0.76	0.82
Egg weight (g)	4	60.50	62.70	61.84	62.01	0.30	0.05	0.25	0.14
	8	62.74	64.06	62.90	64.92	0.48	0.34	0.17	0.37
	12	59.46	61.66	61.18	61.99	0.49	0.27	0.29	0.35
Albumen	4	5.86	5.86	5.93	6.45	0.17	0.60	0.20	0.38
Height (mm)	8	6.85	7.11	7.28	7.64	0.14	0.24	0.04	0.12
	12	6.33	6.35	6.91	7.53	0.19	0.06	<0.01	0.02
Yolk color	4	5.70	5.57	5.80	6.03	0.12	0.57	0.21	0.41
	8	5.96	6.47	6.07	6.39	0.13	0.45	0.42	0.71
	12	5.59	4.93	5.48	5.56	0.10	0.05	0.49	0.32
HU	4	74.21	71.35	73.39	76.92	1.56	0.68	0.39	0.51
	8	81.20	82.51	84.25	85.58	0.81	0.24	0.04	0.12
	12	75.5	76.37	81.39	85.45	1.55	0.05	<0.01	0.02

a *HU, Haught unit.*

b *P-value: A represents one-way ANOVA and Duncan's multiple comparisons; L and Q represent linear and quadratic analysis using regression analysis, respectively*.

**Table 5:** Effects of dietary UPro on albumen quality of laying hens.

**Items**	**UPro levels (% intake)**				**SEM**	***P*** -value^*^		
	**0**	**0.2**	**0.4**	**0.8**		**A**	**L**	**Q**
Albumen weight (g)	38.20	40.65	38.80	40.22	0.50	0.27	0.25	0.48
Thick albumen weight (g)	22.08	22.60	22.74	23.45	0.36	0.63	0.41	0.60
Albumen proportion (%)	64.78	65.50	64.85	65.64	0.25	0.54	0.35	0.64
Water content (g)	90.78	92.78	92.06	91.27	0.88	0.88	0.99	0.79
Crude protein content (%)	84.73^a^	85.23^ab^	85.91^b^	85.95^b^	0.18	0.04	0.01	0.02

a, b *Means within a row with no common superscripts differ significantly (P < 0.05).*

* *P-value: A represents one-way ANOVA and Duncan's multiple comparisons; L and Q represent linear and quadratic analysis using regression analysis, respectively*.

### Texture Properties of Heat-Induced Albumen Gel

The effects of UPro on protein gel properties of albumen gel are presented in Table 6. No significant differences (*P* > 0.05) were noted in cohesiveness and springiness in response to dietary UPro addition. Significant differences in hardness, adhesiveness, and chewiness were observed between dietary treatments (*P* < 0.05), which reduced linearly and quadratically in dietary treatments with the dietary UPro supplementation increasing (*P* < 0.05).

**Table 6:** Effect of dietary UPro on protein gel properties.

**Items**	**UPro levels (% intake)**				**SEM**	***P*** -value^*^		
	**0**	**0.2**	**0.4**	**0.8**		**A**	**L**	**Q**
Hardness (N)	5.92^ab^	6.70^b^	5.65^ab^	5.01^a^	0.20	0.03	0.02	0.04
Cohesiveness	0.59	0.61	0.59	0.59	0.006	0.70	0.66	0.76
Springiness (mm)	3.94	3.93	3.94	3.95	0.03	1.00	0.86	0.98
Gumminess (N)	4.27^ab^	4.85^b^	4.13^ab^	3.56^a^	0.16	0.03	0.02	0.03
Chewiness (mJ)	16.79^ab^	19.03^b^	16.27^ab^	13.96^a^	0.61	0.03	0.02	0.03

a, b *Means within a row with no common superscripts differ significantly (P < 0.05).*

* *P-value: A represents one-way ANOVA and Duncan's multiple comparisons; L and Q represent linear and quadratic analysis using regression analysis, respectively*.

### Plasma Biochemistry

The plasma parameters of laying hens at week 4, 8, and 12 of the experimental periods are shown in Table 7. No significant differences among all groups for the concentration of ALT, AST, ALP, TB, TP, ALB, GLU, and UA in serum were detected (*P* > 0.05).

**Table 7:** Effects of dietary UPro on serum indexes of laying hens.

**Items^**a**^**	**Time (w)**	**UPro levels (% intake)**				**SEM**	***P*** **-value** ^**b**^		
		**0**	**0.2**	**0.4**	**0.8**		**A**	**L**	**Q**
TB (U/L)	4	3.74	4.08	2.90	3.90	0.20	0.15	0.93	0.48
	8	4.32	2.60	4.80	3.80	0.30	0.37	0.21	0.34
	12	2.32	1.66	1.86	2.22	0.14	0.33	0.86	0.26
ALT (U/L)	4	5.60	4.60	4.00	3.60	0.40	0.30	0.07	0.15
	8	5.40	2.60	4.80	3.80	0.56	0.32	0.63	0.77
	12	5.80	5.80	4.60	4.20	0.51	0.23	0.67	0.30
AST (U/L)	4	194.20	212.80	186.80	214.60	4.75	0.09	0.30	0.45
	8	215.20	212.80	234.60	214.80	5.67	0.52	0.87	0.59
	12	239.00	263.00	232.30	234.00	5.59	0.19	0.36	0.60
ALP (U/L)	4	271.20	281.20	253.00	435.40	32.41	0.16	0.06	0.08
	8	348.00	330.40	324.40	291.00	32.32	0.95	0.54	0.83
	12	262.20	496.60	298.20	377.80	42.63	0.22	0.72	0.79
TP (g/L)	4	48.75	51.88	45.52	48.75	0.93	0.11	0.58	0.74
	8	50.37	51.12	45.87	53.37	1.45	0.23	0.98	0.16
	12	50.20	51.48	51.40	46.58	1.80	0.40	0.80	0.97
ALB (g/L)	4	20.72	20.85	19.73	20.62	0.28	0.53	0.75	0.61
	8	21.37	20.88	20.08	21.94	0.37	0.37	0.57	0.23
	12	20.77	18.30	19.99	19.64	0.41	0.20	0.73	0.58
GLU (mmol/L)	4	6.31	6.78	8.19	6.48	0.33	0.16	0.80	0.13
	8	6.41	7.19	5.61	5.85	0.32	0.33	0.30	0.59
	12	8.44	9.00	8.26	8.68	0.23	0.73	0.96	1.00
UA (U/L)	4	186.00	174.40	162.40	160.20	9.16	0.77	0.32	0.60
	8	234.80	193.20	195.40	215.40	11.57	0.56	0.74	0.39
	12	162.00	157.60	164.80	139.60	6.96	0.61	0.27	0.45

a *TB, bilirubin; ALT, alanine aminotransferase; AST, aspartate aminotransferase; ALP, alkaline phosphatase; TP, total protein; ALB, albumin; GLU, glucose; UA, uric acid.*

b *P-value: A represents one-way ANOVA and Duncan's multiple comparisons; L and Q represent linear and quadratic analysis using regression analysis, respectively*.

### Histomorphology of Ileum and Jejunum

**Figure 1:**
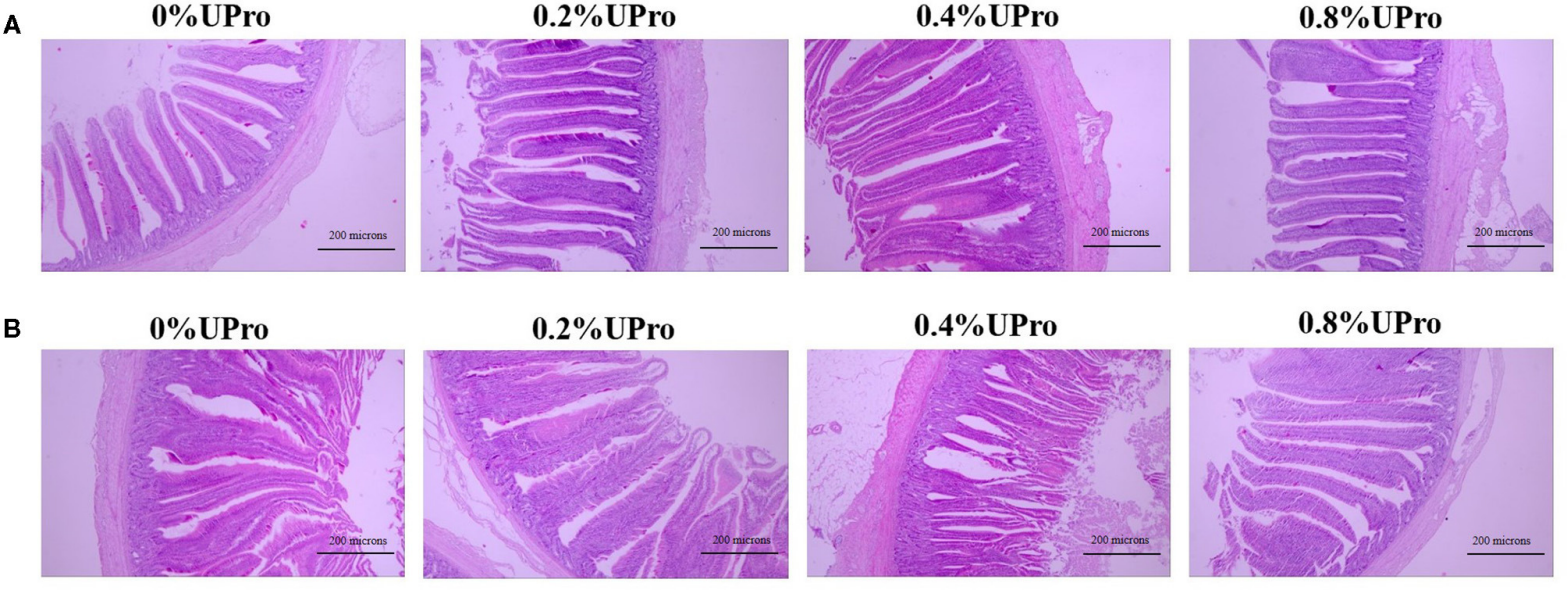
Effects of dietary UPro on intestinal histomorphology of laying hens (40 × ). **(A,B)** The representative pictures of ileal and jejunum sections stained with hematoxylin and erosion (H&E), respectively.

The effect of dietary UPro supplementation on intestinal morphology of laying hens is shown in Table 8. The representative picture of intestinal paraffin sections of each group is shown in Figure 1. UPro supplementation had no significant influences on VH and VCR of ileum (*P* > 0.05). However, the CD of ileum responded a quadratic change (*P* < 0.05), that is, it was first decreased and then increased with the level of UPro supplementation increasing, leading to the shallowest CD in the 0.4% UPro supplemented group. No significant effects were detected in CD of jejunum responding to UPro supplementation (*P* > 0.05). Nevertheless, there were significant changes in VH and VCR of jejunum among all the groups (*P* < 0.05). VH and VCR of jejunum increased linearly along with the UPro supplementation (*P* < 0.05), and VCR of the jejunum also increased quadratically (*P* < 0.05).

**Table 8:** Effect of feeding UPro on intestinal morphology of laying hens.

**Items^**#**^**	**UPro levels (% intake)**				***P*** -value^*^		
	**0**	**0.2**	**0.4**	**0.8**	**A**	**L**	**Q**
**Ileum**							
Villus height/μm	896.83	943.25	906.59	1032.38	0.21	0.06	0.15
Crypt depth/μm	198.73	186.91	187.86	214.52	0.06	0.09	0.03
V/C	4.52	5.00	4.83	4.81	0.11	0.39	0.17
**Jejunum**							
Villus height/μm	1293.2^a^	1405.79^ab^	1319.52^ab^	1444.44^b^	0.04	0.04	0.13
Crypt depth/μm	276.66	302.54	292.71	283.33	0.20	0.99	0.21
V/C	4.67^a^	4.67^a^	4.55^a^	5.15^b^	<0.01	0.01	<0.01

a, b *Means within a row with no common superscripts differ significantly (P < 0.05).*

# *V/C, the villus height/crypt depth.*

* *P-value: A represents o one-way ANOVA and Duncan's multiple comparisons; L and Q represent linear and quadratic analysis using regression analysis, respectively*.

### Gene Expression of the Magnum

**Figure 2:**
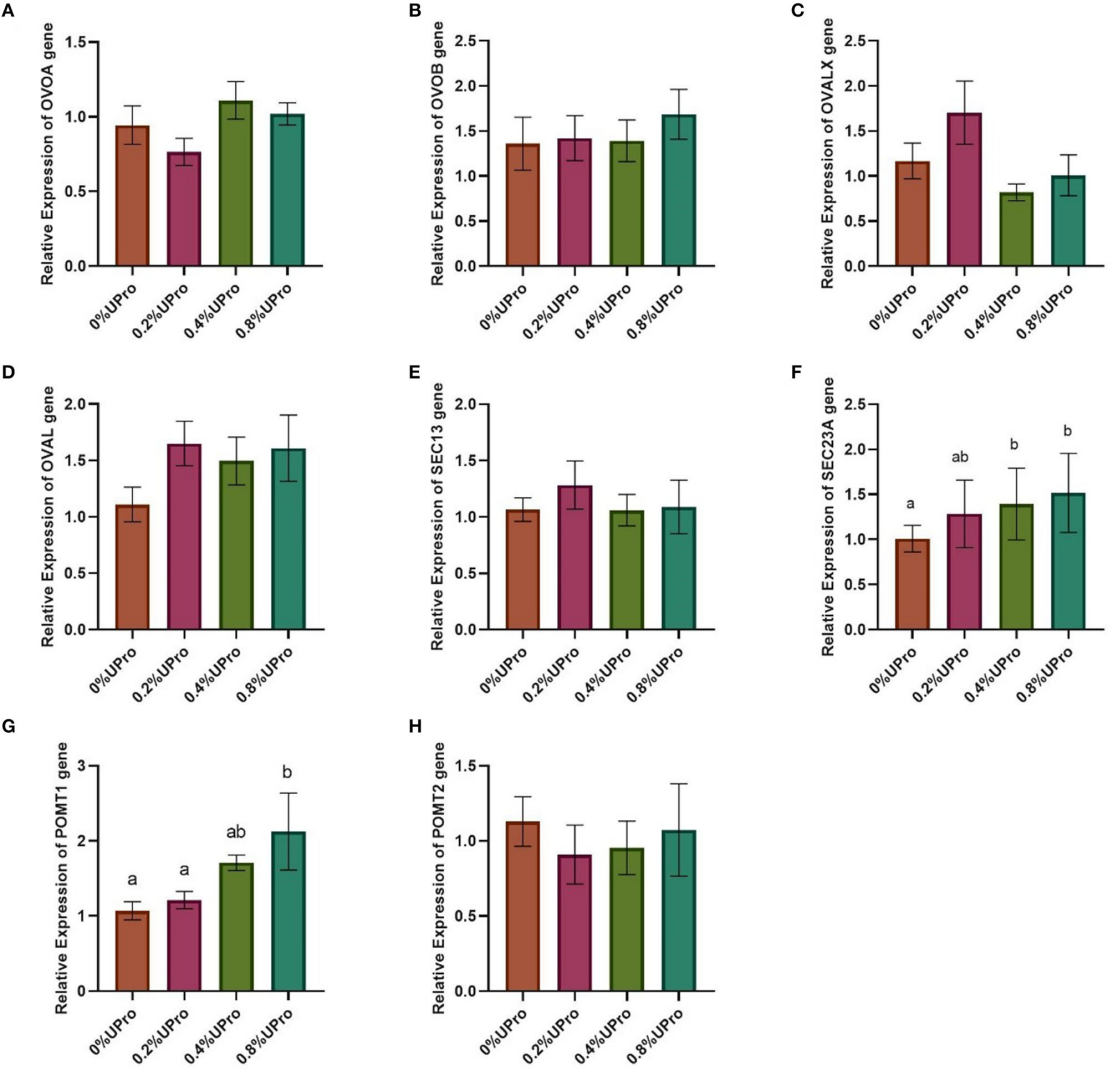
Effects of UPro levels on the relative expression of different genes in the magnum of laying hens. Samples were determined by qPCR. β-actin was regarded as the reference genes. **(A,B,C and D)** genes related to protein synthesis (*OVOA*, ovomucin, alpha subunit; *OVOB*, ovomucin, beta subunit; *OVALX*, ovalbumin-related protein X; *OVAL*, ovalbumin); **(E,F)** genes related to protein secretion (*Sec13*, Sec13 homolog, nuclear pore and COPII coat complex component; *Sec23A*, Sec23 homolog A, COPII coat complex component); **(G,H)** Genes related to protein glycosylation (*POMT1*, protein-O-mannosyltransferase 1; *POMT2*, protein-O-mannosyltransferase 2). The bars with no common letters significantly differ (*P* < 0.05).

No significant changes (*P* > 0.05) in the relative mRNA expression of *OVOA, OVOB, OVALX, OVAL, Sec13*, or *POMT2* in the magnum of laying hens responded to dietary UPro addition (Figure 2). Compared with the control, the relative gene expression of POMT1 in the magnum of laying hens was increased significantly with the UPro addition increasing (*P* < 0.05). And the gene expression of *POMT1* increased linearly and quadratically along with the UPro supplementation (*P* < 0.05). Except for the differences between 0 and 0.2% UPro groups, which were not statistically significant, the differences in the other groups were statistically significant. The relative mRNA expression of *POMT1* in the 0.8% UPro group was the highest. The gene expression of *Sec23A* exhibited a linear and quadratic increase in response to the increasing addition of UPro (*P* < 0.05). Except for the differences between 0.4 and 0.8% UPro groups, which were not statistically significant, the differences in the other groups were statistically significant. The relative mRNA expression of *Sec23A* in the 0.8% UPro groups was the highest.

## Discussion

Albumen is a transparent colloidal solution containing protein, which is secreted by epithelial cells in the magnum of the laying hens (34). It is the most easily available animal-derived protein with high quality (35). The low quality of albumen on the market today affects the vital interests of consumers and producers (36). Because protein source feeds affect the quality of albumen, regulating the quality of albumen through feeds is of great significance to the development of eggs in the future (3, 4). Therefore, UPro enriched with small peptides are worthy of being used as a feed additive to improve the quality of albumen in laying hens.

Small peptides are divided into two categories according to their functions, namely functional peptides and nutritive peptides. Nutritive peptides provide a nitrogen frame and promote protein synthesis. Functional peptides, such as antibacterial peptides, have special effects (37). Certain decomposition products of small peptides regulated physiological functions, which increased the absorption rate of animals and improved production performance (15). Previous reports evidenced that small peptides improved the production performance indexes of laying hens such as egg production and feed conversion rates (38). In the present study, UPro addition had no significant effects on the production performance of the laying hens. The types and spatial distribution of amino acids lead to small peptides with many complex structures, and small peptides effectively improved animal production performance which mainly depends on their molecular structure (39). Herein, the inconsistency may be caused by different molecular structures of small peptides.

With regard to egg quality of layers, this study observed a linear elevation in albumen height, HU, and crude protein content of albumen in reaction to the increase of dietary UPro addition at the 12th week. Similarly, researchers observed that adding appropriate small peptides significantly increased the strength of eggshell and HU in response to the increasing addition of small peptides (40). Moreover, small peptides are related to the deposition of protein and small peptides addition could gradually improve the quality of egg whites, which could explain why linear and quadratic significances occur in 12 weeks instead of over the entire trial period (41). It was previously evidenced that small peptides' addition elevated the freshness of eggs by improving the nutrient absorption capacity of the gastrointestinal tract, which, ultimately, ameliorated the health of the poultry (42). Most digestive processes of nutrients exist in the small intestine, where the height and density of small intestinal villi are able to determine the absorption of nutrients. It was proven that the more time chyme was in contact with the intestinal villi for, the more nutrients would be absorbed (43, 44). A declined CD and an improved VCR suggest the enhancement in the absorption function of the small intestine (45). Consistent with the previous studies (46–48), UPro addition in this study significantly increased VH and VCR of jejunum, which indicated that the absorption of UPro was conducive to the development of intestinal mucosal epithelial cells in laying hens, stimulated intestinal hormone receptors, and promoted the development of the small intestine. Further, it was reported that small peptides' addition not only increased the activity of aminopeptidase in the intestine, but also increased the activity of dipeptidase (49). Therefore, UPro addition improved intestinal health and function, which may further improve the health state and egg quality of laying hens.

The biochemical indicators of blood are essential parameters for judging the homeostasis of the animal's internal environment and the degree of animal health (50). The activity of ALT is considered as an indicator related to changes in the liver functions and can reflect the protein in the body metabolism. ALT activity is low under normal conditions of animals; when liver parenchyma is damaged, ALT activity will increase (51). In the current study, UPro reduced ALT concentration of laying hens, suggesting that UPro might have a protective effect on the liver and can reduce protein metabolism efficiency. The liver is one of the most important organs in animals, and most of the blood in the liver comes from the intestine, but at the same time, the bile produced by the liver flows back into the intestine and directly affects the health of the intestine. It can be seen that the liver has the most direct relationship with the intestines. No significant changes were noted in the TP level in respond to UPro addition; it was different from the results of previous studies (52), where small peptides augmented TP level in the blood. The reason for the difference may be that UPro addition reduced ALT activity, resulting in no significant increase in protein efficiency, so no significant changes were observed in the TP level. According to the findings, UPro addition improved the health of laying hens by ameliorating intestinal health.

The gel properties of albumen are essential for eggs that are widely used in food production. The hardness and chewiness of the gel characteristics reflected the taste of egg white and were used to evaluate the quality of eggs (53). There were many reports on how to affect the characteristics of egg white gel, most of which studied the effect of external processing on albumen (54). However, there were few reports on the improvement of albumen gel properties of eggs by dietary nutritional strategies in laying hens. In the present study, UPro addition significantly reduced the hardness, adhesiveness, and chewiness of the albumen. There was a negative correlation between the Haugh unit and the gel properties (55), and dietary UPro supplementation significantly increased the HU of albumen. Therefore, it may be a useful nutritional method to improve the taste and processing characteristics of albumen by adding UPro to diets.

The magnum is a glandular tissue where the epithelial cells secrete and transport molecules together to synthesize ovalbumin (56). During the first few hours of the ovulation cycle, the egg is in the magnum, during which the protein stored in the epithelium is secreted into the lumen (57). In the later stage of the ovulation cycle, after the egg leaves the magnum, the processes of protein synthesis and modification start immediately (58–60). In the present study, the genes related to the synthesis [*OVOA, OVOB, OVALX*, and *OVAL*, (24)], secretion [*Sec13* and *Sec23A*, (25)], and glycosylation [*POMT1* and *POMT2*, (26)] of protein were selected for the exploration of mechanism underlying the positive effects of UPro on albumen quality. In the process of protein transport from the endoplasmic reticulum to the Golgi apparatus, COP II can wrap the endoplasmic reticulum secretion vesicles and transport secreted proteins from the rough endoplasmic reticulum to the Golgi apparatus (61–63). Sec13 and Sec23 are parts of the coat protein complex COP II (25, 64). Sec23 is a GTPase activating protein, which promotes the separation of target vesicles; Sec13 has an architectural effect and promotes the transportation process (65). Sec13 can also be used as a component of the nuclear pore complex to promote the transport of all nucleoplasm (66). POMT1 and POMT2 distribute in the endoplasmic reticulum membrane, which can synthesize O-mannan by catalyzing the protein-O-mannosylation reaction lines (67, 68). O-mannan is one of the most common methods of post-translational modification in eukaryotes. Protein-O-mannosylation plays a vital role in multiple complex physiological processes in organisms (69). In the current study, UPro showed no effects on the gene expression of *OVO* and *OVA* and increased the gene expression of *Sec23A* and *POMT1*. No changes were observed in the gene expression of *Sec13* in response to UPro addition, which may partially demonstrate that UPro separates target vesicles by stimulating the activity of GTP enzyme, thereby promoting protein transport. Therefore, it could be deduced that UPro improves albumen quality probably through up-regulating the secretion and glycosylation pathways, not the synthesis pathway of egg white proteins, that is, by increasing the gene expression of *Sec23A* and *POMT1* to regulate the formation of albumen. Since there are no related reports currently about the function of *POMT1* in laying hens, the in-depth mechanism of *POMT1* regulating egg quality needs further study.

## Conclusion

UPro addition from 0 to 0.8% in the laying hens' diet dose-dependently improved the intestinal health, enhanced the absorption of nutrients, and then improved albumen quality by increasing the albumen height and HU and decreasing the hardness, stickiness, and chewiness of albumen of eggs. The optimal added amount of UPro should be determined based on further study together with maximized economic benefits. UPro improves albumen quality probably through up-regulating the secretion and glycosylation pathways of egg white proteins by increasing the gene expression of *Sec23A* and *POMT1* in magnum. These findings may contribute to the production of high-quality eggs through the new nutritional additive, UPro, and provide new ideas for exploring the molecular mechanism to improve albumen quality.

## Data Availability Statement

The raw data supporting the conclusions of this article will be made available by the authors, without undue reservation.

## Ethics Statement

The animal protocol was approved by the Animal Care and Use Committee of the Feed Research Institute of Chinese Academy of Agricultural Sciences.

## Author Contributions

XC performed animal experiments, analyzed the data, and wrote the manuscript. SW and KQ contributed to the experimental design and the revision of manuscript. JW, HZ, and GQ provided continuous guidance and assisted with data analysis. SY and SM supervised for the experiment. All authors approved the manuscript.

## Conflict of Interest

SY and SM were employed by Changzhou Yayuan Biochemical Technology Co., Ltd. Changzhou Yayuan Biochemical Technology Co., Ltd provided the samples of UPro used in this study. The remaining authors declare that the research was conducted in the absence of any commercial or financial relationships that could be construed as a potential conflict of interest.

## Publisher's Note

All claims expressed in this article are solely those of the authors and do not necessarily represent those of their affiliated organizations, or those of the publisher, the editors and the reviewers. Any product that may be evaluated in this article, or claim that may be made by its manufacturer, is not guaranteed or endorsed by the publisher.
